# Role of primary cilia in non-dividing and post-mitotic cells

**DOI:** 10.1007/s00441-017-2599-7

**Published:** 2017-03-30

**Authors:** Gerd Walz

**Affiliations:** Renal Division, Department of Medicine, University Freiburg Medical Center, Hugstetter Strasse 55, 79106 Freiburg, Germany

**Keywords:** Ciliopathy, DNA damage response, Autophagy, mTOR, Mitochondria

## Abstract

The essential role of primary (non-motile) cilia during the development of multi-cellular tissues and organs is well established and is underlined by severe disease manifestations caused by mutations in cilia-associated molecules that are collectively termed ciliopathies. However, the role of primary cilia in non-dividing and terminally differentiated, post-mitotic cells is less well understood. Although the prevention of cells from re-entering the cell cycle may represent a major chore, primary cilia have recently been linked to DNA damage responses, autophagy and mitochondria. Given this connectivity, primary cilia in non-dividing cells are well positioned to form a signaling hub outside of the nucleus. Such a center could integrate information to initiate responses and to maintain cellular homeostasis if cell survival is jeopardized. These more discrete functions may remain undetected until differentiated cells are confronted with emergencies.

## Introduction

Cilia are microtubular organelles that control essential signaling pathways during embryogenesis. Disruption of the functional or structural integrity of cilia results in characteristic developmental defects collectively termed ciliopathies. Primary non-motile cilia are often compared with antennae that integrate stimuli from the extracellular environment to initiate cellular signaling. However, with the exception of photoreceptors and olfactory neurons (Falk et al. 2015), the signals recorded by cilia have remained largely elusive. Furthermore, although their role during development is unambiguous, the function of primary cilia in differentiated tissues and in adulthood is not well understood. One of the few noticeable phenotypes after the genetic deletion of cilia in adult mice is hyperphagia and adiposity, supposedly resulting from the defective regulation of satiety responses in pro-opiomelanocortin-expressing neurons (Davenport et al. 2007). Other investigators have demonstrated that the elimination of cilia in the kidneys soon after birth results in no remarkable defect or a slowly progressive cystic phenotype (Lantinga-van Leeuwen et al. 2007; Patel et al. 2008; Piontek et al. 2007). These observations question the functions of cilia in non-dividing and post-mitotic cells, perhaps with the exception of the janitorial tasks of motile cilia.

The intimate relationship between cilia and the cell cycle is unsurprising, since cilia and mitotic spindles are microtubule-based and both use components of the same molecular machinery. Whereas the mitotic spindle is essential for cell cycle progression and cytokinesis, cilia supposedly prevent cells from entering the cell cycle. However, if this were their only task, then cilia would be completely superfluous, for example, on terminally differentiated cells that lack the ability to re-enter the cell cycle.

Based on recent findings, cilia might hypothetically sense nutritional status, energy supply, cell shape, cell volume and cell polarity, in addition to damaging events that compromise cell survival and integrate this information to prevent senescence and/or cell death. If cilia play such a role, they need to orchestrate responses after (nuclear or mitochondrial) DNA damage, for example, caused by hypoxia-induced release of reactive oxygen species (ROS), to activate autophagy in situations of nutritional deprivation, or to initiate apoptosis if irreversible damage has occurred. The overarching decision to live or die certainly requires a central hub rather than multi-focal, uncoordinated and potentially antagonistic subsidiaries. It may make sense to place such a center outside of the nucleus, since replication stress is not an issue in non-dividing cells. Emerging observations suggest that cilia can play such a fundamental role if cellular emergencies arise. However, if everything goes well, the function of cilia in non-dividing cells may remain concealed. Cilia in non-dividing cells may also be involved in more subtle phenotypes, for example, the flow-dependent regulation of potassium secretion (Carrisoza-Gaytan et al. 2016).

## Cilia and the cell cycle

With few exceptions (Paridaen et al. 2013), cilia are disassembled in most dividing mammalian cells during G1/S phase. The basal body and the attached daughter centriole revert back to centrioles, elongate during S-G2 phase (Fu et al. 2016) and duplicate to form the mitotic spindle (Nigg and Stearns 2011). An extensive network of negative regulators of ciliogenesis has emerged to ensure ciliary disassembly upon cell cycle re-entry and to prevent cilia formation during cell cycle progression (Izawa et al. 2015; Liang et al. 2016).

In cultured cells, ciliary disassembly and cell proliferation is typically initiated by serum after nutrient deprivation. Upon serum exposure, HEF1 (human enhancer of filamentation-1) binds and activates Aurora A (AurA; Pugacheva et al. 2007). AurA then initiates ciliary disassembly by activating HDAC6, followed by the de-polymerization of tubulin (Plotnikova et al. 2012; Pugacheva et al. 2007). PIFO (pitchfork), a mouse embryonic node gene required for normal left-right asymmetry, accumulates at the basal body and activates AurA to promote ciliary disassembly (Kinzel et al. 2010). WNT5a can stimulate the CK1ε-dependent phosphorylation of DVL2 (Dishevelled-2) to induce interaction with PLK1, which in turn supports HEF1/AurA-mediated ciliary disassembly (Lee et al. 2012). Mitostatin (Trichoplein), a mitochondrial protein with poorly defined oncostatic activities (Cerqua et al. 2010), supports AurA activation in G1 to suppress unscheduled cilia formation (Inoko et al. 2012). NDEL1 (neurodevelopment protein 1 like 1), a modulator of dynein activity, suppresses Mitostatin degradation to inhibit cilia assembly (Inaba et al. 2016).

CP110, recruited to centrosomes by CEP97, suppresses ciliary assembly and the removal of CP110 from the maturing basal body has been postulated to be a prerequisite for ciliogenesis (Spektor et al. 2007), for example, by preventing the interaction of CP110 with CEP290 and by antagonizing the function of CEP290 as a basal-body-tethering molecule (Tsang et al. 2009). High CP110 levels suppress cilia formation, whereas optimal CP110 levels promote ciliogenesis, suggesting that CP110 levels need to be precisely adjusted in dividing and non-dividing cells (Walentek et al. 2016). In proliferating cells, the Joubert syndrone protein inositol polyphosphate-5-phosphatase E (INPP5E) inhibits tau-tubulin kinase-2 (TTBK2) by maintaining phosphatidylinositol 4-phosphate (PI_4_P) levels to prevent the interaction of TTBK2 with CEP164 and ciliogenesis (Xu et al. 2016).

The anaphase-promoting-CDC20 complex is required for the timely resorption of the cilium after serum stimulation, thereby regulating the stability of microtubules through the targeting of NEK1 for degradation (Wang et al. 2014). The dynein light chain DYNLT1 (Tctex-1) promotes ciliary disassembly before S phase and has been shown to prevent radial glia cells from prematurely exiting the cell cycle in the developing neocortex (Li et al. 2011). Several kinesins support ciliary disassembly (Hu et al. 2015). The kinesin-13 family member KIF2A has microtubule-depolymerizing properties and is activated by PLK1 (Miyamoto et al. 2015); PLK1 is highly expressed during G2/M and plays important roles in centrosome maturation, spindle assembly and cytokinesis (Zitouni et al. 2014).

CEP76, which prevents centriole reduplication during the cell cycle (Tsang et al. 2009), is a CDK2 (cyclin-dependent kinase 2) substrate and is phosphorylated by CDK2 to prevent the activation of PLK1 (Barbelanne et al. 2016). NDE1 (nuclear distribution gene E homolog 1), acting upstream of the dynein light chain DYNLL1 (LC8), is a negative regulator of ciliary length and is highly expressed in M phase (S. Kim et al. 2011). Depletion of NDE1 in embryonic rat brains causes cell cycle arrest of neural progenitor cells and severe microcephaly (Doobin et al. 2016) consistent with a role of NDE1 in maintaining replicating progenitor cells. Fidgetin-like-1 (FIGL-1) is a recently identified centrosomal protein that prevents ciliogenesis (Zhao et al. 2016). Interestingly, this AAA-ATPase has also been implicated in DNA repair responses (Girard et al. 2015; Yuan and Chen 2013).

Although ciliary length might influence G1 duration (S. Kim et al. 2011; Wang et al. 2015), the organelle itself appears to prevent cells from entering the cell cycle (for a review, see Goto et al. 2016). The observation that forced ciliogenesis can inhibit cell cycle progression suggests that cilia act as a checkpoint prior to cell cycle re-entry (Inoko et al. 2012). To permit ciliogenesis, inhibitors are removed upon cell cycle exit by proteasomal degradation or by autophagy. NDE1 is degraded by CDK5-SCF (Maskey et al. 2015). Mitostatin is degraded by the Cul3-Ring E3 ligase (CRL3)-KCTD17 complex (Kasahara et al. 2014). MST1/2, a component of the Hippo pathway, phosphorylates AurA and dissociates the AurA/HDAC6 cilia-disassembly complex to promote ciliogenesis (M. Kim et al. 2014).

During S-phase progression, centrioles are duplicated exactly once. The CDK1/Cyclin B complex binds STIL (SCL/TAL1 interrupting locus) in mitosis to prevent its interaction with PLK4 (Polo-like kinase 4), which together with STIL and SAS-6 (spindle assembly abnormal protein 6) forms a core module for centriole duplication (Arquint et al. 2015; Arquint and Nigg 2016). PLK4 phosphorylates STIL in G1, promoting centriole biogenesis during S phase (Zitouni et al. 2016). Mutations in PLK4, STIL and SAS-6 result in primary microcephaly, a disease manifestation that is also observed in patients with mutations in proteins involved in DNA damage responses (see below).

**Fig. 1:**
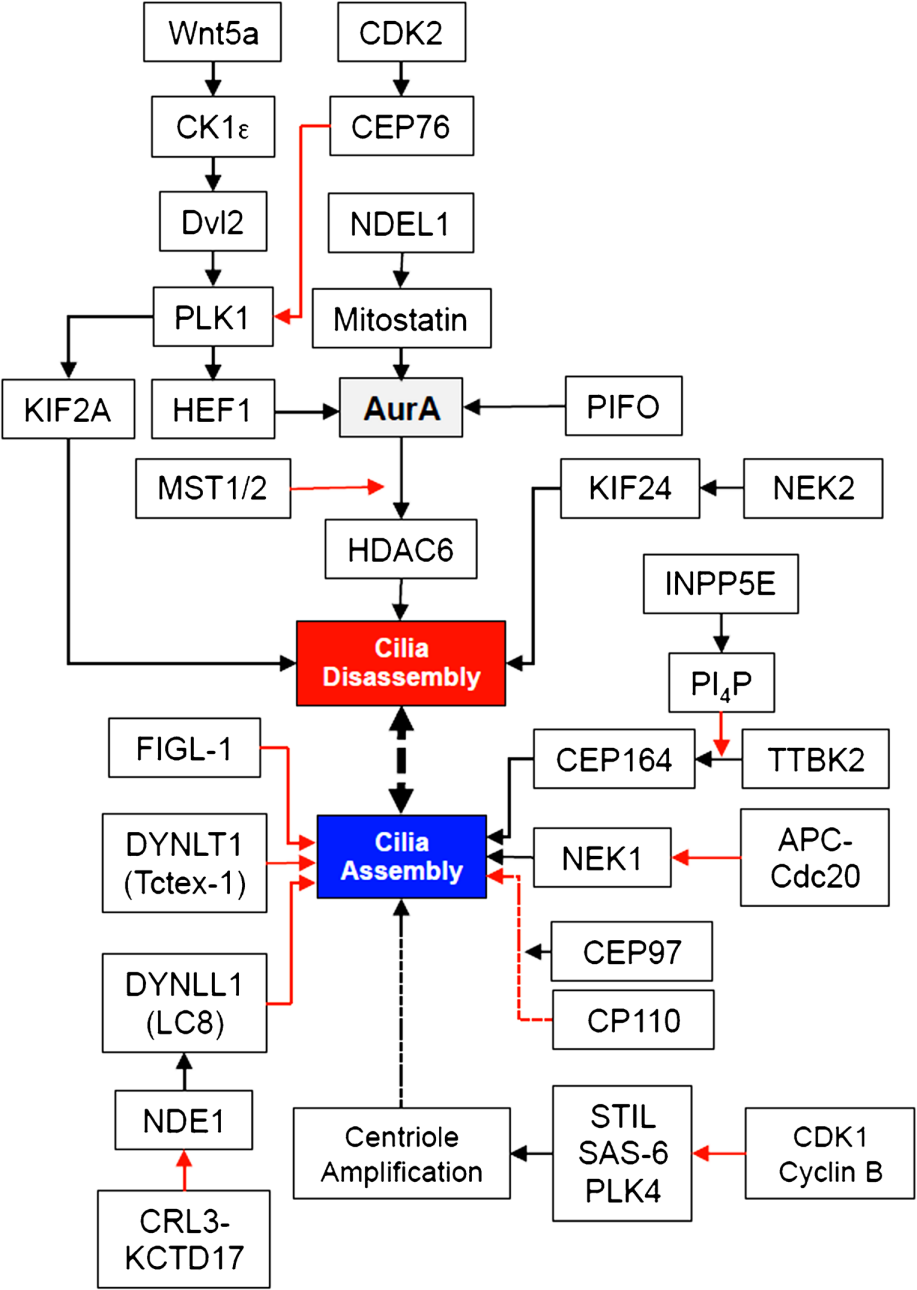
Proteins involved in cilia assembly and disassembly. This representation is an over-simplification of a dynamic process that controls (*bottom*) cilia assembly in cells exiting the cell-cycle and (*top*) cilia disassembly in proliferating cells. HEF1 activates Aurora A (AurA), which initiates ciliary disassembly by activating HDAC6. PIFO promotes ciliary disassembly, supporting AurA activation. Mitostatin supports AurA activation; its degradation is inhibited by NDEL1. Wnt5a-mediated activation of CK1e triggers phosphorylation of Dvl2 and interaction with PLK1, which in turn favors stabilization of HEF1. KIF2A, a kinesin family member that depolymerizes microtubules, is phosphorylated and activated by PLK1. INPP5E maintains PI4P levels to prevent the interaction of TTBK2 with CEP164 and ciliogenesis. APC-Cdc20 targets NEK1 for degradation, whereas Nek1 supports the stability and integrity of cilia. CEP97 recruits CP110 to the centrosome; CP110 suppresses ciliary assembly at high levels, whereas optimal levels of CP110 are required for ciliogenesis. DYNLT1 promotes ciliary disassembly before S phase. Control of centriole duplication is essential to prevent premature ciliogenesis. CDK1/Cyclin B bind STIL to prevent the premature formation of the STIL/SAS-6/PLK4 complex required for centriole duplication. CEP76 is phosphorylated by CDK2 to prevent the activation of PLK1 and centriole reduplication during the cell cycle. NDE1 acts upstream of DYNNL1 (LC8) and negatively controls ciliary length. The MSt1/2 component of the Hippo signaling cascade promotes ciliogenesis by dissociating the AurA/HDAC6 cilia-disassembly complex. NEK2 facilitates centriole separation but phosphorylates KIF24 to promote ciliary disassembly (see text for abbreviated protein names and further details). Note that the depicted interactions do not occur simultaneously but are precisely coordinated with the cell cycle (*black lines* indicate positive, *red lines* negative regulation).

Thus, multiple signaling components prevent ciliogenesis during the cell cycle, whereas other molecules have evolved to stabilize cilia in non-dividing cells (Fig. 1). The balance between ciliary assembly and disassembly is cell-cycle-dependent and controlled by molecules that coordinate centriole duplication and formation of the mitotic spindle apparatus.

## Cilia in cell growth and cancer

Cilia play important roles in cell signaling pathways that control cell proliferation, differentiation and cell size and polarity, including the Wnt, mTOR and Hippo signaling pathways. The Wnt signaling cascades, broadly categorized into a canonical β-catenin-dependent and a non-canonical β-catenin-independent branch, intersect in multiple ways with cilia (for a review, see Oh and Katsanis 2013). Although Dishevelled, a Wnt core protein is required for the formation and polarization of motile cilia (Park et al. 2008), cilia-associated molecules of the nephronophthisis (NPHP; Bergmann et al. 2008; Burckle et al. 2011; Simons et al. 2005) and Bardet-Biedl syndrome (BBS; May-Simera et al. 2015; Ross et al. 2005) gene families antagonize canonical Wnt and promote non-canonical planar cell polarity signaling (for a review, see Goggolidou 2014).

Two pathways antagonistically influence cell growth and cell shape, namely the mTORC1 and Hippo signaling cascades; not surprisingly, both pathways are linked to cilia. Flow activates ciliary LKB1/AMPK signaling to suppress mTOR activation via the TSC1/TSC2 complex (Boehlke et al. 2010) and controls cell volume through the regulation of autophagy (Orhon et al. 2016). mTOR activity is frequently increased in cells lacking intact cilia (Ibraghimov-Beskrovnaya and Natoli 2011). However, ciliary defects do not necessarily result in increased proliferation. For example, mutant Arl13b, responsible for Joubert syndrome, is associated with the reduced proliferation of fibroblasts (Pruski et al. 2016) and the elimination of cilia only marginally increases the proliferation of renal epithelial cells in adult murine kidneys (Piontek et al. 2007). The Hippo signaling cascade controls cell proliferation, survival and differentiation by phosphorylating the transcriptional co-activators YAP and TAZ, curtailing their nuclear activity through cytoplasmic retention and degradation (for a review, see Hansen et al. 2015). The regulatory serine-threonine kinase module MST1 (mammalian STE20-like protein kinase 1) and MST2, together with their activating adaptor protein SAV1 (Salvador family WW domain-containing protein 1), interact with nephrocystins (NPHPs) at the transition zone and promote ciliogenesis by interfering with AurA/HDAC6 activity (M. Kim et al. 2014). Surprisingly, members of the NPHPs induce YAP/TAZ activity, facilitating cell growth. Whereas NPHP4 binds to LATS to prevent the inhibition of YAP/TAZ (Habbig et al. 2011), NPHP3/NPHP9 release TAZ from 14-3-3 binding and cytoplasmic retention (Habbig et al. 2012). Future research needs to reconcile these apparently opposing signals generated by cilia, suppressing TORC1 but augmenting YAP/TAZ-mediated growth signaling.

Since cilia act as gatekeepers to prevent cell cycle re-entry, they are unsurprisingly absent from several cancer types (Basten and Giles 2013; Seeger-Nukpezah et al. 2013). Furthermore, important tumor-promoting pathways are linked to cilia, including the Sonic Hedgehog (Shh), LKB1, Wnt and Hippo pathway. However, some cancers also display increased numbers of primary cilia (Yasar et al. 2016), suggesting that the role of cilia in tumor cells depends on the oncogenic driver and the cancer type. Examples of the ambiguous role of cilia in cancer development include oncogenic mutations of Shh signaling components. In the absence of Shh, Gli2 and Gli3 are cleaved within the ciliary compartment to generate transcriptional repressors (for a review, see Bangs and Anderson 2016). After Shh-mediated activation, Smoothened accumulates in the ciliary compartment to generate active Gli2 and Gli3 that translocate to the nucleus and trigger the transcription of Shh target genes. Basal cell carcinomas, induced by an activated form of Smoothened, benefit from the presence of cilia and are suppressed by ciliary ablation, whereas the loss of cilia and lack of Gli2/Gli3 repressor molecules are advantageous for tumors caused by constitutively active Gli2 (Han et al. 2009; Wong et al. 2009).

Mutations of the Von Hippel-Lindau (VHL) tumor suppressor result in multiple benign and metastatic tumors, including renal cell cancer. VHL, which targets HIF1α for degradation, promotes ciliogenesis by stabilizing microtubules (Hergovich et al. 2003; Schermer et al. 2006). However, deletion of VHL has not consistently been associated with the loss of cilia and knockout of VHL does not cause RCC in mice; indeed, the combined deletion of three genes, namely VHL, Trp53 and Kif3a, is required to cause cystic and neoplastic lesions (Albers et al. 2013; Guinot et al. 2016). Nevertheless, knockout of VHL alone results in disorganized cilia and aberrant cell proliferation reminiscent of renal clear cell cancer in zebrafish, supporting a more direct involvement of VHL in ciliogenesis and cell-cycle control in this vertebrate model (Noonan et al. 2016). Of note is the connection between VHL and oxygen tension. Under normal oxygen levels, prolyl-hydroxylated HIF1α is targeted for proteasomal degradation by VHL (Hsu 2012). Other ciliary proteins responsive to oxygen tension are Anks3 and Anks6 (NPHP16). Both proteins are asparagine-hydroxylated at specific sites within their ankyrin-repeat domains by HIF1α inhibitor (HIF1AN; Hoff et al. 2013; Yakulov et al. 2015). These findings suggest that cilia respond to changes in oxygen tension; however, the significance of these oxygen-dependent post-translational modifications is currently unknown.

## Cilia and DNA damage response

Informative phenotypes have led to the observation that cilia-associated molecules (CEP164) can participate in DNA damage responses (DDR), whereas mutations of molecules involved in DDR (MRE11, ZNF423) can produce ciliopathy phenotypes (Chaki et al. 2012). Normal neurogenesis depends on the rapid expansion of neural progenitor cells, requiring error-free DNA replication and efficient cell division. Hence, mutations of centrosomal proteins (e.g., pericentrin, CEP152), components of the mitotic spindle apparatus, or DDR machinery are often associated with microcephaly (for a review, see Alcantara and O’Driscoll 2014). Although cerebellar abnormalities and vermis aplasia are characteristic of certain ciliopathies (e.g., nephronophthisis and Joubert syndrome), microcephaly and an increased cancer risk associated with defective DNA repair and genome instability of proliferating cells are usually not observed in patients with ciliopathies. However, mutations in Origin Licensing Proteins (*ORC1*, *ORC4*, *ORC6*, *CDT1* and *CDC6*) cause Meier-Gorlin syndrome, characterized by microcephaly, dwarfism and ear and skeletal abnormalities; this syndrome is also not linked to faulty cell cycle progression but to defective cilia formation and Shh signaling (Bicknell et al. 2011; Stiff et al. 2013).

Three kinases, namely Ataxia Telangiectasia Mutated (ATM), Ataxia Telangiectasia, and Rad3-related (ATR) and DNA Protein Kinase (DNA-PK) together with members of the poly-ADP-ribose polymerase (PARP) proteins sense DNA damage and initiate repair responses. ATM and DNA-PK are primarily activated by DNA double-strand breaks (DSB), whereas ATR and PARPs are mostly activated by stretches of single-strand DNA occurring at stalled replication forks (Cimprich and Cortez 2008).

**Fig. 2:**
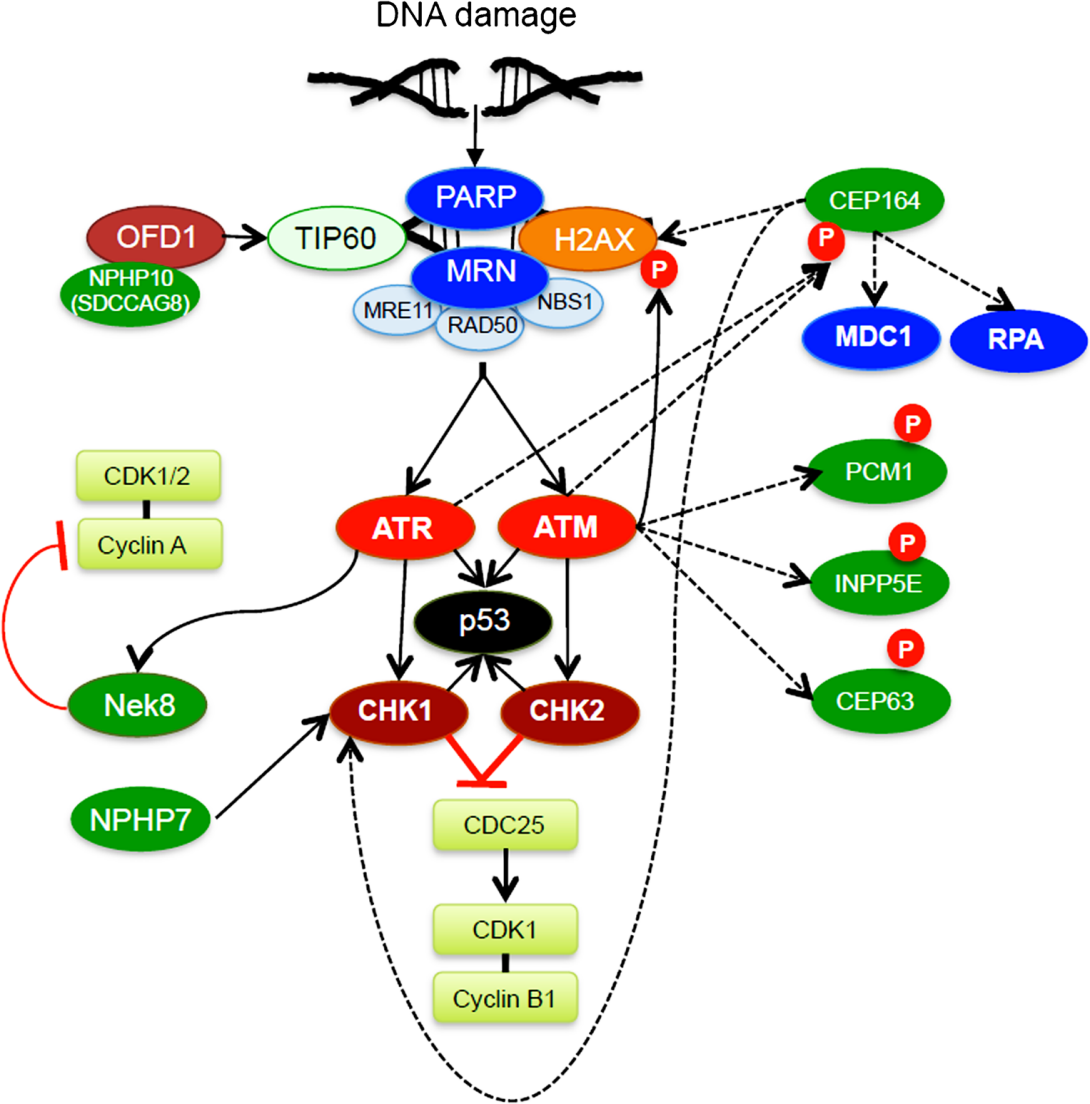
Representation of cilia-associated molecules that have been implicated in DNA damage responses (DDR). DNA damage recruits members of the poly-ADP-ribose polymerase (PARP) family and the MRN complex (MRE11, RAD40 and NBS1) to induce cell-intrinsic checkpoints, including p53. The ATM/CHK2 module is activated after DNA double-strand breaks (DSB), whereas the ATR/CHK1 pathway responds primarily to DNA signal-strand breaks. Both pathways converge on CDC25 phosphatase, a positive regulator of cell cycle progression upstream of CDK1/Cyclin B1. CEP164 interacts with ATM and ATR and is phosphorylated (*P*) by both kinases. CEP164 facilitates the activation of CHK1, MCD1 and PRA. Nek8 inhibits the cyclin-A-dependent activation of CDK1/2, pausing cell cycle progression. NPHP7 (Glis2) is associated with the activation of checkpoint kinase 1 (CHK1), the stabilization of p53 and the induction of senescence. OFD1 interacts with NPHP10 (SDCCAG8) and components of the TIP60 histone acetyltransferase complex. Cells with defective OFD1 exhibit reduced histone acetylation, impaired repair and prolonged arrest at the G2-M checkpoint after DNA DSB. In response to H_2_O_2_, ATM can undergo auto-phosphorylation to activate LKB1/AMPK and autophagy (for abbreviations, see text)

Several cilia-associated proteins with roles in genome stability have been recently identified, including CEP164, CEP290, NPHP9/NEK8 and NPHP10 (SDCCAG8; Airik et al. 2014; Johnson and Collis 2016). CEP164 interacts with ATM and ATR, is phosphorylated by both kinases in response to DNA damage and promotes the phosphorylation of other DDR components, including RPA (replication protein A), histone H2AX, MDC1 (mediator of DNA damage checkpoint 1) and the serine/threonine kinase CHK1 (Sivasubramaniam et al. 2008; Fig. 2). Other ciliary proteins are phosphorylated by DDR kinases, including ninein, PCM1 (pericentriolar materal 1), INPP5E (inositol polyphosphate-5-phosphatase E) and CEP63 (Matsuoka et al. 2007). The locations at which these interactions and post-translational modifications occur have not been studied in detail; however, several DNA damage proteins localize to the centrosome (Zhang et al. 2007) and ATR has been identified outside of the nucleus in the cilia of photoreceptors (Valdes-Sanchez et al. 2013). Interestingly, reduced *ATR* expression in postnatal mice carrying one mutant *ATR* allele is associated with severe photoreceptor degeneration (Valdes-Sanchez et al. 2013), suggesting that defective DNA repair linked to cilia causes the progressive degeneration of post-mitotic cells. However, the way in which ATR is activated and those substrates that are phosphorylated by ATR outside of the nucleus remain unknown.

Although the *Aspergillus nidulans* NIMA kinase regulates spindle organization, chromosome alignment, cytokinesis and entry into mitosis, the 11 mammalian NIMA-related NEKs have evolved to coordinate microtubule-dependent programs in dividing and non-dividing cells, including centriole disjunction, spindle assembly and ciliogenesis (Fry et al. 2012; Quarmby and Mahjoub 2005). In non-dividing cells, NEK1 has been implicated as a regulator of ciliogenesis (Shalom et al. 2008), potentially through the interaction with KIF3A and the control of microtubular dynamics. NEK1 variants have also recently been described in patients with familial amyotrophic lateral sclerosis (Kenna et al. 2016), a heterogenetic disease linked to alterations in mitochondrial functions and DNA repair. NEK2, which facilitates centriole separation, maintains a balance between ciliogenesis and ciliary disassembly through interaction with the cilia-associated nucleoporin Nup98 (Endicott et al. 2015) and can phosphorylate and activate the microtubule depolymerizing kinesin Kif24 to promote ciliary disassembly independently of the AurA/HDAC6 module (S. Kim et al. 2015). NEK9 is activated by PLK1 and controls centrosome separation through NEK6, NEK7 and Eg5 (Bertran et al. 2011). Loss of NEK9 delays cell cycle progression and has recently been identified as a novel human ciliopathy associated with skeletal dysplasia (Casey et al. 2016). NEK7, which affects microtubule dynamics and is required for mitotic spindle formation, interacts with ANKS3, an ankyrin-repeat protein related to ANKS6 and potential NPHP candidate (Ramachandran et al. 2015; Shamseldin et al. 2016; Yakulov et al. 2015). Knockout of NEK7 in mice leads to lethality in the second half of embryogenesis and is associated with chromosomal lagging, micro-nuclei formation, tetraploidity and ciliary abnormalities (Salem et al. 2010). NEK8 (NPHP9) has recently been identified as a down-stream effector molecule of ATR preventing spontaneous DNA DSB by limiting cyclin-A-dependent kinase (CDK) activity (Choi et al. 2013).

Oral-Facial-Digital syndrome Type I (OFD1) was first described as a component of the TIP60 histone acetyltransferase complex; it localizes to chromatin and to the basal body (Abramowicz et al. 2016). Cells with defective OFD1 exhibit reduced histone acetylation, impaired repair and prolonged arrest at the G2-M checkpoint after DNA DSB. Inactivation of Kif3A results in the loss of primary cilia because of defective intraflagellar transport. Surprisingly, Kif3A-deficient tubular epithelial cells display increased cell proliferation with the loss of cell cycle arrest in response to DNA damage as a result of reduced p53 stability (Lu et al. 2016). In contrast, loss of the nephronophthisis gene product Glis2/NPHP7 is associated with the activation of checkpoint kinase 1 (CHK1), the stabilization of p53 and the induction of senescence, which translates into progressive inflammation, atrophy and fibrosis (Lu et al. 2016).

Ischemia is probably the most prominent insult causing DNA damage in non-dividing and post-mitotic cells. One of the first events following ischemia/reperfusion (IR) injury is a burst of ROS production from mitochondria, probably as a consequence of reverse electron transport and superoxide production by respiratory complex I (Chouchani et al. 2016), resulting in increased phosphorylation of ATM, H2AX, CHK2 and p53 (Ma et al. 2014). In response to H_2_O_2_, ATM can undergo auto-phosphorylation or form an active homo-dimer via intermolecular disulfide bonds. After IR injury, cilia are initially shortened and then lengthened (Verghese et al. 2008, 2009), a process induced by ROS-mediated ERK (extracellular-regulated MAP kinase) activation (J.I. Kim et al. 2013). Cisplatin-induced tubular injury results in the shortening of cilia, whereas tubular epithelial cells with short cilia display increased susceptibility to Cisplatin (Wang et al. 2013). Foxj1, a transcriptional regulator of ciliogenesis, is rapidly up-regulated in response to epithelial cell injury and leads to the induction of *tektin-1*, *dnahc9* and *efhc1* (Hellman et al. 2010). Unilateral nephrectomy is associated with superoxide production in the remaining kidney, causing the lengthening of cilia (Han et al. 2016). The LKB1/AMPK/TSC2 cascade appears to be a target of ATM in response to ischemia, suppressing protein synthesis and inducing autophagy (Ditch and Paull 2012).

The speculation that cilia and cilia-associated molecules are involved in initiating and/or facilitating DNA repair is supported by the role of centrosomes in DNA damage responses (for a review, see Mullee and Morrison 2016). Centrosomes contain DDR components, including ATM, ATR, CHK1/CHK2, BRCA1 and members of the poly(ADP-ribose) polymerase family. Furthermore, centrosomes are strongly affected by DNA-damaging agents, resulting in the distortion of the pericentriolar material and centrosome duplication independent of the cell cycle. The way that DDR proteins are activated outside of the nucleus remains to be elucidated. Recent findings suggest that RNA-binding proteins not only regulate gene expression involved in DDR but also play a direct role in DDR signaling (for a review, see Dutertre and Vagner 2016). Small double-stranded RNAs can be produced by DICER/DROSHA in response to DNA damage and incorporated into Argonaute 2 (AGO2; Wei et al. 2012). AGO2 has been localized to the basal body of astrocytes and knockdown of AGO2 prevents the elongation of the ciliary axoneme (Moser et al. 2011). The bicaudal C homolog 1 (BICC1), an established RNA-binding protein (Rothe et al. 2015), might be a potential candidate recruiting RNA molecules to the centrosome and/or ciliary compartment.

## Cilia and mitochondria

Mitochondria are the main source of ATP in most cells. Both the production and delivery of ATP need to be coordinated, especially in highly polarized cells with different energy requirements across the cell; for example, in neurons, high levels of ATP are required at the synapse in comparison with other cellular compartments. Whereas local mitochondrial movements are accomplished by myosin-mediated transport (myosin V, VI) along short actin filaments, mitochondria can also move along microtubules, utilizing microtubule-based motor and adaptor proteins, to reach subcellular locations with a high-energy demand (for reviews, see Saxton and Hollenbeck 2012; Tang 2016). In neurons, kinesin-1 (Kif5b) and kinesin-3 (Kif1b) mediate the anterograde plus-end-directed transport of mitochondria to the periphery, whereas minus-end-directed dynein motor proteins mediate the retrograde transport of mitochondria back to the cell body. The mitochondria-anchored Rho small GTPase MIRO interacts with the mammalian Milton homologs TRAK1/2, which are two kinesin- and dynein/dynactin-associated proteins that connect mitochondria to microtubule-dependent long-range means of transport. Other MIRO interactors, e.g., mitochondrial fusion factors mitofusion-1 and -2, the PTEN-induced putative kinase 1 (PINK1), HUMMR (Hypoxia Upregulated Mitochondrial Movement Regulator) and ERMES (endoplasmic reticulum [ER]-mitochondrial encounter structure), participate in mitochondrial transport and calcium homeostasis. Since cilia-associated molecules involved in microtubule dynamics are utilized outside of the cilium, ciliary defects might also affect mitochondrial movements.

Although mitochondria are not essential for ciliogenesis (Majumder and Fisk 2013), whether primary cilia consume and are dependent on mitochondrial ATP is unknown. In the flagella of sea urchin sperm, ATP consumption depends on the beating frequency and has been estimated at around 2-3*10^5^ molecules/beat depending on the viscosity of the surrounding fluid; the ATP consumption rate is reduced to approximately 30 % in inactive sperm flagella (Chen et al. 2015). ATP production by glycolysis has been detected within the ciliary axoneme of *Chlamydomonas* flagella and is required for normal motility (Mitchell et al. 2005). However, the energy requirement of primary mammalian cilia remains unknown and the amounts of ATP that are produced by glycolysis within the ciliary axoneme and that are provided by diffusion from the cytoplasm have not been elucidated. Structural analysis of photoreceptors has revealed that mitochondria are present in close proximity of the basal body and connecting cilium, suggesting that the diffusion of ATP into the rod outer segment represents a source of energy supply (Gilliam et al. 2012). Since ATP-consuming motor proteins power intraflagellar transport, ATP deprivation, for example, caused by hypoxia or an insufficient glucose supply, might interfere with ciliary functions. A link between cilia and mitochondria has been suggested by the identification of mutated XPNPEP3, a mitochondrial X-prolyl aminopeptidase, causing nephronophthisis (NPH)-like disease manifestations (Bottinger 2010; O’Toole et al. 2010). Furthermore, Anks6 (NPHP16), a cilia-associated protein mutated in patients with NPH, has been found to interact with several mitochondrial proteins that have been identified by independent mass spectrometry screens, including SAMM50, CHCHD3, Mitofilin, ATP5C1 and VDAC2 (Hoff et al. 2013). Whereas VDAC1 and VDAC3 negatively influence ciliogenesis, VDAC2 localizes to centriolar satellites to facilitate ciliary maturation (Majumder et al. 2015). All three VDAC isoforms have been identified in a non-mitochondrial centrosomal pool, suggesting a function in addition to their established roles in mitochondrial bioenergetics during ciliogenesis.

The ability of cilia to recruit and assemble mitochondria is exemplified in mammalian sperm: OFD2 (outer dense fiber protein 2) recruits Mitostatin (trichoplein), a protein that prevents cilia formation during cell cycle, promotes cilia assembly during quiescence and regulates mitochondrial-ER binding via Mitofusin-2 (MFN2). Mitostatin has also been implicated in the recruitment of Parkin, which interacts with the distress-sensing mitophagic kinase PINK1 (Neill et al. 2015). MNS1 (Meiosis Specific Nuclear Structural 1), which interacts with MFN2 and ODF2, anchors mitochondria peripherally to the (9 + 2) microtubule-containing mid-piece of sperm (Vadnais et al. 2014). CFAP157, a recently identified target gene of FOXJ1 and CEP350-interacting protein, seems to contribute to mitochondrial positioning, since CFAP154-deficient murine sperm display clustering of mitochondria within the sperm mid-piece region (Weidemann et al. 2016). An abnormal accumulation of mitochondria is also observed in the spermatids of IFT20-deficient mice (Zhang et al. 2016), further supporting a link between cilia and mitochondria. Thus, cilia-associated mitochondrial proteins might recruit mitochondrial to the vicinity of basal bodies or connecting cilium to promote ATP delivery to the ciliary axoneme or might coordinate calcium release, mitochondrial depolarization and induction of mitophagy in response to cellular stress. However, close proximity to mitochondria would also render cilia susceptible to ROS produced by mitochondria in response to oxygen deprivation.

## Cilia and autophagy

**Fig. 3:**
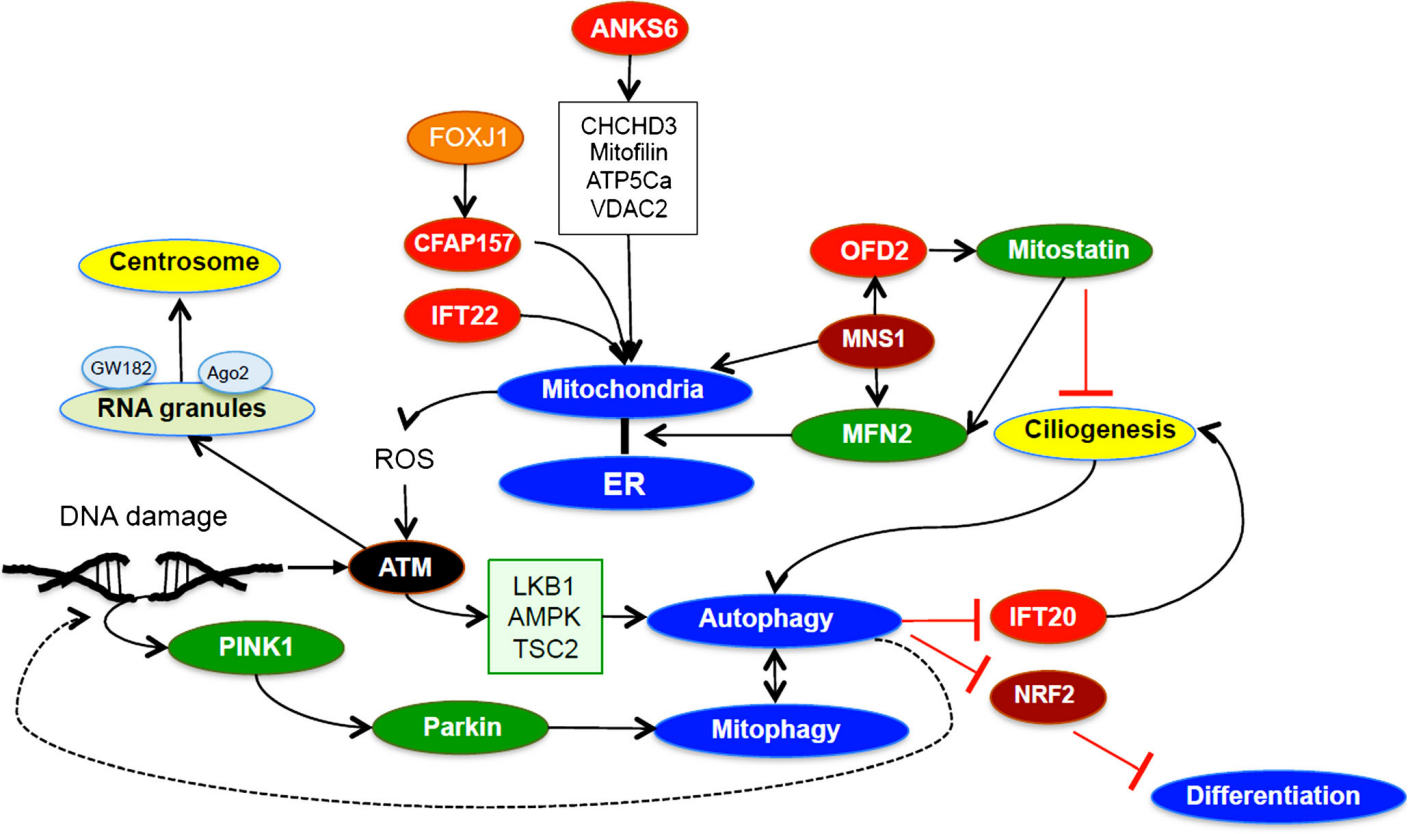
Interactions of cilia with mitochondria, autophagy and differentiation. Anks6 interacts with several mitochondrial proteins; however, the significance of these interactions remains unknown. Mitochondria are not required for ciliogenesis, although ciliary proteins have recently been implicated in mitochondrial positioning in sperm. MNS1, by interacting with MFN2 and ODF2, anchors mitochondria in sperm. CFAP157, a FOXJ1 target gene and IFT22 are also involved in mitochondrial positioning in sperm. OFD2 recruits Mitostatin, which inhibits ciligenesis and regulates mitochondrial-endoplasmic reticulum (ER) contact sites through mitofusin. Autophagy targets IF20 for degradation, preventing ciliogenesis under nutrient-rich conditions. DNA damage can activate LKB1/AMPK/TSC2 via ATM to initiate autophagy. Autophagy in turn might provide the necessary building blocks (e.g., dNTPs) to facilitate DNA repair. Oxidative stress results in ATM activation and stress granule formation. RNA granules, containing GW182 and Ago2, localize to centrosomes or basal bodies of primary cilia. In human embryonal stem cells, cilia activate autophagy to degrade NRF2, promoting neuroectoderm development (for abbreviations, see text)

Mutual relationships between primary cilia and autophagy have recently been reported (for a review, see Pampliega and Cuervo 2016; Fig. 3). Autophagy is a highly conserved pathway for the degradation and recycling of lysosomal cargo. Whereas micro-autophagy delivers cargo to lysosomes by lysosomal membrane invagination, (macro-) autophagy, which is orchestrated by more than a dozen autophagy-related (ATG) proteins, sequesters cargo (including protein aggregates, mitochondria, peroxisomes and lipid droplets) into double-membrane vesicles that are finally targeted to lysosomes (Rubinsztein et al. 2012). Initiation of autophagosome biogenesis requires the activation of an ULK1-containing protein complex, encompassing ATG13, ATG101 and FIP200 (Eliopoulos et al. 2016). Under nutrient-rich conditions, TORC1 prevents ULK1 activation through the phosphorylation of ULK1 at S757; starvation activates AMPK, which phosphorylates ULK1 at S317/S777 and activates TSC2 to inhibit TORC1. Alternative mechanisms of autophagosome formation have been identified; for example, autophagy induced by the deprivation of glucose does not require ULK1/ULK2 (Cheong et al. 2011). Recent findings indicate that autophagy is also activated by DNA damage; for example, ATM can activate AMPK to antagonize the inhibitory effect of TORC1 on autophagy initiation (Alexander et al. 2010) and can trigger AMPK-dependent ULK1 phosphorylation and activation (J. Kim et al. 2011). Damage of either mitochondrial or nuclear DNA can trigger mitophagy. Damaged mitochondria with stress-induced loss of mitochondrial membrane potential fail to import PINK1, which then phosphorylates and activates the ubiquitin ligase Parkin at the outside of mitochondria. Ubiquitination of Parkin-target proteins recruits autophagy adaptor proteins, eventually resulting in mitophagy (Eliopoulos et al. 2016). Defective nuclear DDR results in abnormal mitophagy because of PARP1 hyperactivation, NAD^+^-depletion, impairment of NAD^+^-dependent SIRT1 and diminished expression of the mitochondrial Uncoupling Protein 2 (UCP2; Fang et al. 2014, 2016). By degrading critical components of the DDR pathway and providing essential DNA repair building blocks including dNTPs, autophagy may play a crucial role in supporting the response to DNA damage and has also been implicated in the senescence-associated secretory phenotype (SASP) characterized by the decreased autophagy-dependent degradation of GATA4 and the induction of TRAF3IP2 and interleukin-1α expression (Kang et al. 2015).

Primary cilia with intact anterograde intraflagellar transport and Hedgehog signaling are required for autophagy activation after starvation; this process is characterized by the recruitment of several autophagy-related proteins (ATG) towards the ciliary base (Pampliega et al. 2013). The switch from basal to induced autophagy requires the selective removal of OFD1 from centriolar satellites to promote ciliogenesis (Tang et al. 2013). Under nutrient-rich conditions, basal autophagy prevents ciliary lengthening by degrading IFT20 and spares centriolar OFD1 to suppress ciliogenesis (Tang et al. 2013). Since ciliopathies often increase mTOR activity, the presence of intact cilia seems to shift the balance towards autophagy, keeping mTOR activity under control (Wang et al. 2015). However, whether suppressed autophagy influences disease progression in autosomal dominant polycystic kidney disease, the most common human ciliopathy, is currently unknown.

An important interaction between primary cilia and autophagy has recently been uncovered during neuroectoderm development. Nuclear factor (erythroid-derived 2)-like 2 (NRF2), which is usually targeted for proteasomal degradation by Keap1 (Kelch-like ECH-associated protein 1), coordinates cellular responses to oxidative and electrophile stress by stimulating the expression of genes with antioxidant/electrophile responsive elements (ARE/EpRE) that promote cell survival and restore redox homeostasis (Sihvola and Levonen 2016). In human embryonal stem (ES) cells, NRF2 suppresses neuroectoderm development by controlling OCT4 and NANOG expression. Neuroectoderm-specific lengthening of the G1 phase allows primary cilia to activate autophagy. Autophagy-dependent degradation of NRF2 results in the differentiation of ES into neuroectoderm (Jang et al. 2016). Cilia-dependent autophagy may play a similar role in terminating proliferative responses in the kidney during development or after injury. Interestingly, cilia ablation in the adult kidney has no obvious consequences; however, if cilia-deficient renal tubular cells are forced to proliferate after ischemia-induced necrosis, a rapid proliferation-triggered onset of cyst formation occurs (Patel et al. 2008), supporting the notion that cilia play an essential role in putting the brake on proliferation and in promoting differentiation. Furthermore, cilia might control antioxidant and survival responses through the regulation of NRF2.

In addition to DDR, a close link also exists between ROS signaling and autophagy (for a review, see Filomeni et al. 2015). Superoxide has been suggested as being the molecule that is produced in response to starvation and that is responsible for autophagy induction (Chen et al. 2009). Overburdened mitochondria, resulting in increased ROS production, might shift the role of ROS as an inducer of autophagy to an inducer of mitophagy as a negative feedback mechanism to remove mitochondria as the source of oxidative stress.

## Cilia and stress granules

Approximately 800 RNA-binding proteins (RBPs) control RNA metabolism from transcription, splicing, nuclear export, translation, to degradation. RNA-protein interactions have also been implicated in the regulation of enzymatic and non-enzymatic activities of bound proteins, whereas metabolites can compete with mRNA to control metabolic pathways. Only about half of these proteins contain a canonical RNA-binding domain that bind specific recognition elements usually located in the 3′ untranslated region (Mitchell and Parker 2014). In response to stress, RNA-binding proteins translocate from the nucleus to the cytoplasm, where they form aggregates with capped mRNA (stress granules [SGs]) to switch ribosomal synthesis from highly specialized to protective housekeeping proteins (Wolozin 2014). The formation of large macro-molecular, sometimes detergent-insoluble complexes is facilitated by the prion-like low sequence complexity and high glycine content of RNA-binding proteins (Han et al. 2012; Kato et al. 2012). Among the many types of RNA granules, processing bodies (PBs) and SGs are the best-characterized (Anderson et al. 2015). PBs are enriched for factors involved in mRNA processing and degradation, including exo-ribonuclease XRN1, decapping enzymes DCP1/DCP2, deadenylase complex CCR4/CAF1/NOT, nonsense-mediated mRNA decay proteins UPF1-3, LSM1-7, DDX6 and SMG5-7 and components of the RNAi machinery (e.g., GW182 and Argonautes); however, less then 10 % of these factors are concentrated in PBs. SGs encompass translation factors, 40S ribosomal subunits, diverse RNA-binding proteins associated with stalled mRNAs and G3BP1 (Ras-GTPase-Activating Protein SH3-Domain-Binding Protein 1). The formation of SGs is initiated by events that result in the phosphorylation of eukaryotic initiation factor 2 (eIF2) and the inhibition of translational initiation. Arsenite is a potent inducer of SG formation by causing oxidative stress (Anderson et al. 2015). G3BP1 and the Ubiquitin-specific protease 10 (USP10) coordinately regulate antioxidant activities in response to arsenite, involving ATM activity (Takahashi et al. 2013). The stability of USP10 is controlled by the autophagy component Beclin1 (Liu et al. 2011), establishing complex interactions between oxidative stress, SG formation and autophagy.

Two P bodies, containing GW182 and Ago2, specifically localize to the centrosome or the basal body of primary cilia in cultures of human astrocytes and synoviocytes (Moser et al. 2011); they are required for normal ciliogenesis, supporting a link between RNA-containing granules and cilia (Fig. 3). Furthermore, a recent short interfering RNA screen for genes essential for ciliogenesis has identified a number of RNA-modifying proteins, including PRPF31, LSM2 and LSM5, which are also present in P bodies (Wheway et al. 2015).

## Concluding remarks

Primary cilia decorate both non-dividing cells that can re-enter the cell cycle and terminally differentiated cells that lack this ability. Since primary cilia in post-mitotic cells are superfluous for preventing cell cycle re-entry, the question arises as to whether cilia exert any functions in these cells. Indeed, adult life does not reveal an essential role for primary cilia, except in situations in which cells are confronted with stress compromising cell survival. Cells that re-enter the cell cycle to replace necrotic or apoptotic cells, for example, after ischemia or toxic injuries, seem to require primary cilia to terminate proliferative responses and/or to re-assume tissue-specific functions. Examples are human ES cells that generate primary cilia to escape re-cycling and to differentiate into neuroectoderm (Jang et al. 2016) or tubular epithelial cells that rapidly form cysts after ischemic injury in the absence of primary cilia (Patel et al. 2008). In cells that cannot respond to injury with proliferation to replace dead tissue, the function of primary cilia may be more discreet. To prevent tissue degeneration through progressive cell loss or forced senescence, primary cilia may sense and direct repair responses to ensure cell survival. The speculation that primary cilia form a signaling hub to respond to insults that jeopardize cellular integrity is supported by their intricate involvement in crucial molecular programs that maintain cellular homeostasis. Recent observations link cilia-associated molecules to DNA damage responses, autophagy and mitochondria. Conceptually, a signaling hub that lies outside of the nucleus and that integrates nutritional status, energy supply and exposure to damaging events might possibly coordinate appropriate responses more effectively in non-dividing cells in which replication-induced DNA damage is not concern. Cilia are also involved in the regulation of mTOR and Hippo signaling; these signaling cascades need to be coordinated to prevent the activation of antagonistic cellular programs and responses. Whereas ciliogenesis and ciliary disassembly has been extensively studied resulting in the identification of a myriad of molecules, future work needs to focus on the function of cilia in cellular homeostasis. This may require in vivo approaches or primary cell cultures to circumvent the limitations posed by the proliferating cancer cells that are typically used to delineate the function of primary cilia.
